# Trends in mortality due to acute myocardial infarction and pneumonia among older adults in the U.S.: a CDC wonder analysis (1999-2023)

**DOI:** 10.3389/fcvm.2026.1913867

**Published:** 2026-09-30

**Authors:** Shuai Feng, Lianghao Zhang, Lin Quan, Nannan Jiang, Ziyi Zhao, Hongxiang Ji, Chuanshen Xu, Jinquan Liu

**Affiliations:** 1 Organ Transplantation Center, The Affiliated Hospital of Qingdao University, Qingdao, Shandong, China; 2 Department of Orthopaedics, The Affiliated Hospital of Qingdao University, Qingdao, Shandong, China; 3 Department of Hand and Foot, Microsurgery, The Affiliated Hospital of Qingdao University, Qingdao, Shandong, China; 4 The First Clinical Medical College, Shandong University of Traditional Chinese Medicine, Jinan, Shandong, China

**Keywords:** acute myocardial infarction, CDC WONDER, mortality trends, older adults, pneumonia

## Abstract

**Objective:**

This study aims to assess the comprehensive national trends in deaths related to pneumonia and AMI in the US from 1999 to 2023.

**Methods:**

CDC wonder multiple cause of death database was used to extract data of elderly (≥65 years) with pneumonia and AMI as main or contributing cause of death. Age-Adjusted Mortality Rates (AAMRs) per 100,000 population were calculated. Trends were stratified by sex, race/ethnicity, state, urban-rural classification, census region, ten-year age group, and place of death.

**Results:**

A total of 186,411 deaths associated with pneumonia and AMI during 1999-2023 were identified. The cumulative AAMR was 17.76 during 1999-2020, and 14.2 during 2021-2023. A significant decline in AAMR was found through the study period (AAPC:-4.26, 95% CI −6.71 to -1.74). The AAMR of men was higher than women, and the decrease of AAMR was steeper in women (AAPC:-4.38, 95% CI -6.78 to -1.92 among women vs. -4.07, 95% CI -5.68 to -2.43 among men). NH American Indian or Alaska Native has the highest AAMR, while NH Asian or Pacific Islander had the lowest AAPC (-5.03, 95% CI -7.95 to -2.02). Non-metropolitan had higher AAMR (22.93 vs. 16.59) and AAPC (-1.69 vs. -3.51) than metropolitan. South region showed the highest AAMR and AAPC compared to other census regions.

**Conclusions:**

The AAMR per 100,000 of pneumonia and AMI decreased among older adults in the US during 1999-2023, with higher mortality burden among men, NH American Indian or Alaska Native, rural area, and South region.

## 1 Introduction

Acute myocardial infarction (AMI) arises when the blood supply to a section of the heart is severely diminished or obstructed, resulting in damage and ultimately the necrosis of heart muscle cells. This obstruction is commonly due to the accumulation of atherosclerotic plaque or, in certain instances, a spasm of the coronary arteries (1). In the United States, cardiovascular disease (CVD) continues to be the primary cause of death, with its age-adjusted mortality rate (AAMR) approximately twice that of cancer (2). AMI itself is responsible for more than half of these cardiovascular-related fatalities, particularly affecting individuals aged 65 and older (3). It is estimated that the number of people aged 65 years and older would reach 80 million at the time of 2040 (4). Therefore, AMI-related research is receiving ever-growing attention.

Pneumonia is an infection of the lung parenchyma caused by bacteria, viruses, fungi, or parasites, leading to considerable local and systemic inflammation. It is listed as one of the top ten causes of death for American, and responsible for more than 53,000 deaths and 2.6 million visits to emergency departments in the US in 2020 (5, 6). Besides, Pneumonia is a major contributor to mortality and functional deterioration in the elderly population (7). A retrospective study of 1,949,352 adults aged ≥65 years showed that community-acquired pneumonia imposes a greater burden in terms of hospitalizations and overall costs compared to myocardial infarction, stroke, and osteoporotic fractures (8). The diagnosis and treatment of pneumonia has been an important issue worldwide.

Patients with pneumonia are prone to AMI, which further leads to poor prognosis. A self-controlled case series study included 13,494 adults from National Health Service hospitals across Portugal during 2010-2018, and demonstrated an increased risk of myocardial infarction for community-acquired pneumonia patients (9). After examining 2 community-based cohorts, Corrales-Medina et al. concluded that hospitalization for pneumonia was associated with increased short-term and long-term risk of cardiovascular disease (10). Given the close clinical interrelationship between pneumonia and AMI, examining deaths in which both conditions are co-reported may reveal distinct temporal and subgroup mortality patterns that are not apparent when either condition is analyzed separately.

Although previous studies have investigated the national mortality patterns of AMI and pneumonia among American separately (11, 12), no study has focused on the mortality trend of patients with both AMI and pneumonia in the US. This study aims to comprehensively analyze the nationwide temporal trends and demographic disparities of deaths associated with AMI and pneumonia among the US elderly between 1999 and 2023. Such an evaluation would provide a valuable basis for public health policy and the identification of at-risk populations.

## 2 Method

### 2.1 Study design

We performed a cross-sectional time-trend analysis using mortality data obtained from the Centers for Disease Control and Prevention (CDC) Wide-ranging Online Data for Epidemiologic Research (WONDER) platform (13). This publicly available system compiles death certificate data for all U.S. residents. The analysis covered the period from 1999 to 2023, during which we assessed mortality trends among adults aged 65 years and older who had AMI and pneumonia.

The study reviewed death records from the Multiple Causes of Death Public Use Registry to identify instances where AMI and pneumonia were either a contributing factor or the primary cause of death. The diagnostic codes were I21 for AMI and J12-J16, J18, J69 for pneumonia to systematically and comprehensively reflect relevant death, as per the International Statistical Classification of Diseases and Related Health Problems, Tenth Revision (ICD-10), in line with prior research (14, 15). Additionally, a sensitivity analysis was conducted for deaths where AMI was the primary cause, with pneumonia listed as a contributing factor.

As this study utilized publicly accessible and fully de-identified data, approval from an institutional review board (IRB) was not necessary. The research adhered to the STROBE guidelines for cross-sectional observational studies.

### 2.2 Data extraction

This study includes population size, year, demographic characteristics, location of death, regional classification, state-specific data, and urban-rural categorization.

Demographic encompassed age, sex, and race/ethnicity. Age groups were defined as 65–74, 75–84, and 85 + years, while race/ethnicity categories included non-hispanic (NH) White, NH Black or African American, NH American Indian or Alaska Native, NH Asian or Pacific Islander, and Hispanic or Latino. The location of death was categorized into Medical Facility, Decedent's Home, Hospice Facility, Nursing Home/Long Term Care, and Other. According to the criteria established by the U.S. Census Bureau, the Regional divisions were divided into four primary geographical categories: Northeast, Midwest, South, and West regions (16). Based on 2013 National Center for Health Statistics Urban–Rural Classification Scheme for Counties, the urbanization status was defined as metropolitan for counties with populations ≥ 50,000, and non-metropolitan for those with populations < 50,000 (17).

### 2.3 Statistical analysis

Crude and age-adjusted mortality rates (AAMRs) per 100,000 individuals from 1999 to 2023 were calculated to examine regional trends in AMI and pneumonia-related mortality, with breakdowns by year of death, sex, race/ethnicity, state, and urban/rural status. A 95% Confidence Interval (CI) was applied. To calculate the crude mortality rate, the total number of AMI and pneumonia-related deaths was divided by the corresponding U.S. population for each year. AAMR was determined by standardizing AMI and pneumonia-related deaths to the U.S. population of 2000 (18). As CDC wonder database provide the Multiple Cause of Death Data in 1999-2020 and 2018-2023, the overall AAMR assessment was presented in two segment (1999-2020 and 2021-2023), and the 2021–2023 data were extracted from the 2018–2023 dataset (19, 20). Annual percent changes (APCs), average annual percent changes (AAPCs), and their corresponding 95% Cis were calculated using the Joinpoint Regression Program (Version 5.2.0.0, National Cancer Institute) (21). This study aimed to analyze the annual trends in AMI and pneumonia-related deaths by employing log-linear regression models to identify significant changes in AAMR over time. The APCs were considered to show an increase or decrease when the slope reflecting changes in mortality was significantly different from zero, as determined by 2-tailed t-tests. The AAPC was calculated as a weighted average of the segment-specific log-linear slope coefficients, with weights proportional to the duration of each segment, and was subsequently back-transformed to an annual percentage change. R statistical software version 4.3.0 (R Project for Statistical Computing) was used for data visualization.

## 3 Result

### 3.1 Overall trends

From 1999 to 2023, a total of 186,411 deaths associated with AMI and pneumonia were identified among U.S. adults aged ≥65 years. The overall AAMR per 100,000 population was 27.63 (95% CI 27.07 to 28.18) in 1999, and 10.67 (95% CI 10.39 to 10.95) in 2023, with the cumulative AAMR per 100,000 of 17.76 (95% CI 17.67 to 17.85) between 1999 and 2020, and 14.2 (95% CI 14.02 to 14.39) between 2021 and 2023 (Table 1; Supplementary Tables S1, S2).

**Table 1:** Demographic characteristics of death associated with acute myocardial infarction and pneumonia among the elderly in the USA between 1999 and 2023.

Variables	Deaths (%)	AAMRs (95% CI) per 100,000 1999-2020	AAMRs (95% CI) per 100,000 2021-2023
Overall	186411 (100%)	17.76 (17.67–17.85)	14.2 (14.02–14.39)
Sex			
Female	87,176 (46.77%)	13.64 (13.54–13.73)	10.13 (9.93–10.34)
Male	99,235 (53.23%)	24.18 (24.02–24.34)	19.99 (19.64–20.33)
Race			
NH White	153,302 (82.45%)	18.01 (17.91–18.11)	14.25 (14.04–14.47)
NH Black or African American	14,279 (7.68%)	16.63 (16.34–16.93)	13.68 (13.06–14.3)
NH Asian or Pacific Islander	6,032 (3.24%)	16.15 (15.7–16.6)	12.75 (11.96–13.53)
NH American Indian or Alaska Native	950 (0.51%)	19.67 (18.28–21.07)	16.53 (13.88–19.17)
Hispanic or Latino	11,361 (6.11%)	15.78 (15.45–16.11)	15.56 (14.9–16.22)
Age groups^a^			
65–74 years	35,683 (19.14%)	5.77 (5.7–5.83)	6.12 (5.97–6.27)
75–84 years	67,769 (36.35%)	19.94 (19.78–20.1)	15.85 (15.51–16.19)
85 + years	82,959 (44.5%)	62.54 (62.09–62.99)	44.05 (43.1–45.01)
Census Region			
Northeast	36,029 (19.33%)	17.43 (17.24–17.62)	10.47 (10.1–10.84)
Midwest	42,166 (22.62%)	17.73 (17.55–17.91)	13.42 (13.02–13.81)
South	69,445 (37.25%)	18.14 (17.99–18.29)	16.31 (15.99–16.64)
West	38,771 (20.8%)	17.3 (17.12–17.49)	14.57 (14.17–14.97)
Urbanization (1999–2020)			
Metropolitan	125,686 (76.79%)	16.59 (16.5–16.69)	NA
Nonmetropolitan	37,996 (23.21%)	22.93 (22.69–23.16)	NA
Place of death			
Medical Facility	153,741 (82.65%)	NA	NA
Decedent's Home	7,537 (4.05%)	NA	NA
Hospice Facility	4,033 (2.17%)	NA	NA
Nursing Home/Long Term Care	18,716 (10.06%)	NA	NA
Other	1,994 (1.07%)	NA	NA

a Crude mortality rates are used for age groups.

The AAMR decreased from 1999 to 2018 (APC:-5.28, 95% CI -5.77 to -4.80, *p* < 0.001), and increased insignificantly from 2018 to 2021 (APC:19.27, 95% CI -0.72 to 43.29, *p* = 0.059), subsequently declined steeply between 2021 and 2023 (APC:-23.73, 95% CI -36.63 to -8.20, *p* = 0.007). In general, the AAMR showed a decreased trend through the study period (AAPC:-4.26, 95% CI -6.71 to -1.74, *p* = 0.001)(Figure 1; Supplementary Figure S1).

**Figure 1:**
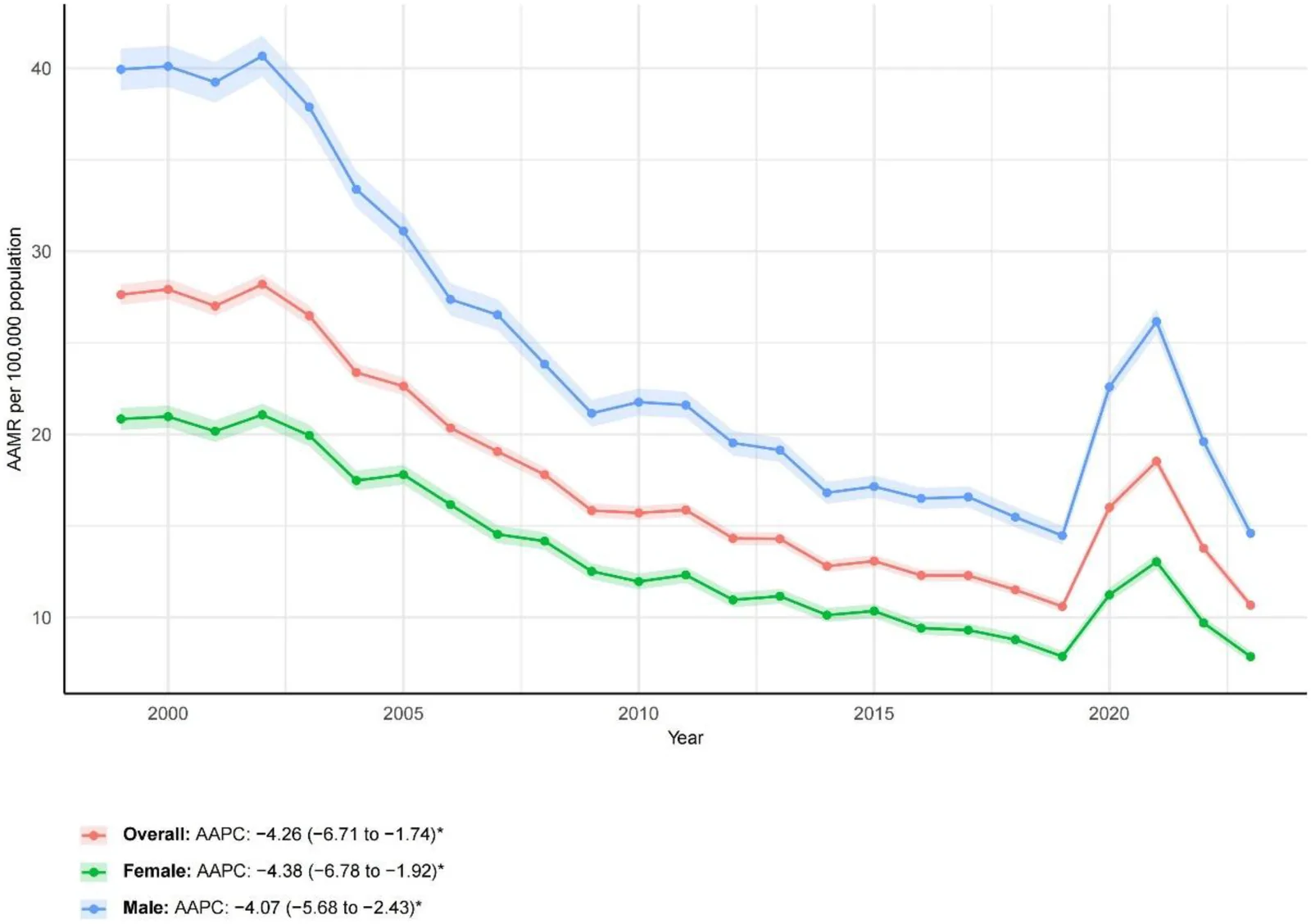
Overall and sex-stratified AAMRs per 100,000 associated with AMI and pneumonia in the US, 1999 to 2023 * indicates that the AAPC is significant.the shaded area represents the 95% CI. AAMR, age-adjusted mortality rate; AMI, acute myocardial infarction; AAPC, average annual percent changes; CI, confidence interval.

### 3.2 Trends by sex

There were 99,235 deaths in older men (53.23%), which was higher than that of women with 87,176 deaths (46.77%). The AAMRs per 100,000 were higher in men compared to women both in 1999-2020 period (24.18, 95% CI 24.02 to 24.34 vs. 13.64, 95% CI 13.54 to 13.73) and 2021-2023 period (19.99, 95% CI 19.64 to 20.33 vs. 10.13, 95% CI 9.93 to 10.34)(Table 1; Supplementary Tables S1, S2).

The AAMR of both men and women decreased between 1999 and 2018, with a steady decline during 1999-2018 in women (APC:-5.11, 95% CI -5.56 to -4.66, *p* < 0.001), and a steep decline during 2002-2008 (APC:-8.57, 95% CI -10.64 to -6.45, *p* < 0.001) followed by a mild decline during 2008-2018 (APC:-4.57, 95% CI -5.60 to -3.54, *p* < 0.001) in men. From 2018 to 2021, there was an upward trend in both men (APC:20.58, 95% CI 8.16 to 34.42, *p* = 0.003) and women (APC:14.43, 95% CI -4.23 to 36.72, *p* = 0.129), but only significant among men. Between 2021 and 2023, men (APC:-24.85, 95% CI -32.78 to -15.99, *p* < 0.001) and women (APC:-21.42, 95% CI -34.72 to -5.41, *p* = 0.014) had a similar and rapid decrease of AAMR. Generally, the decline of AAMR is slightly steeper in women (AAPC:-4.38, 95% CI -6.78 to -1.92, *p* < 0.001) than men (AAPC:-4.07, 95% CI -5.68 to -2.43, *p* < 0.001)(Figure 1; Supplementary Figure S1).

### 3.3 Trends by race/ethnicity

The NH White contribute to most of the deaths (82.45%), followed by NH Black or African American (7.68%) and Hispanic or Latino (6.11%). However, NH American Indian or Alaska Native has the highest AAMR per 100,000 both in 1999-2020 period (19.67, 95% CI 18.28 to 21.07) and 2021-2023 period (16.53, 95% CI 13.88 to 19.17)(Table 1; Supplementary Tables S1, S3).

NH White, NH Black or African American,Hispanic or Latino, and NH Asian or Pacific Islander had similar change in AAMR through the study period, with significant decrease during 1999-2017/2018 and 2021-2023. The significant increases during 2017/2018-2021 were only found in Hispanic or Latino and NH Black or African American. Notably, NH American Indian or Alaska Native showed a continuous decline during the whole study period. Totally, there was a significant decrease in all race, among which NH Asian or Pacific Islander had the lowest AAPC (-5.03, 95% CI -7.95 to -2.02, *p* = 0.001), followed by NH Black or African American(AAPC:-4.24, 95% CI -6.11 to -2.35, *p* < 0.001), NH White(AAPC:-4.17, 95% CI -6.76 to -1.50, *p* = 0.002), Hispanic or Latino(AAPC:-4.11, 95% CI -7.18 to -0.94, *p* = 0.011), and NH American Indian or Alaska Native(AAPC:-2.67, 95% CI -4.22 to -1.08, *p* = 0.002)(Figure 2; Supplementary Figure S2).

**Figure 2:**
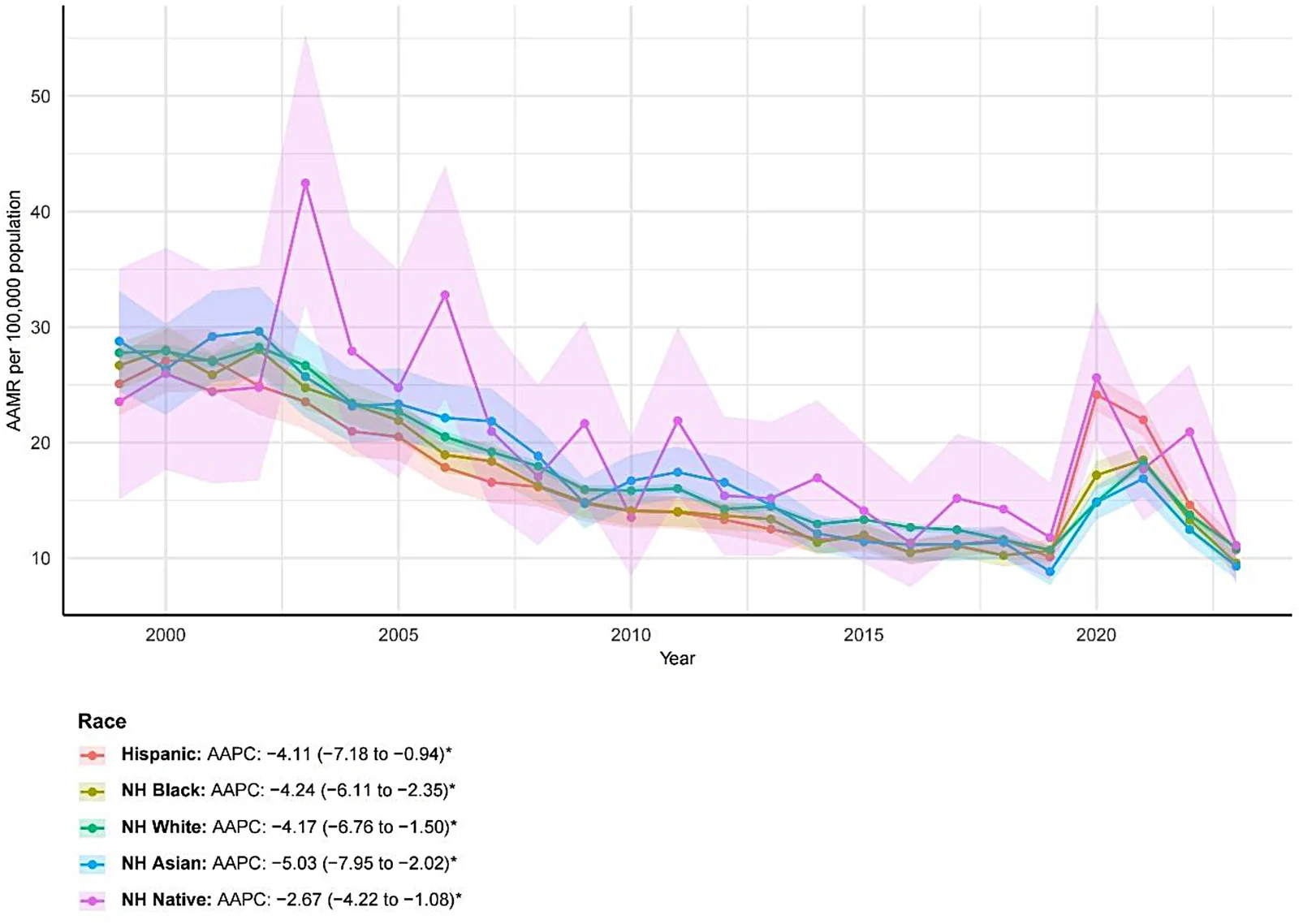
Race-stratified AAMRs per 100,000 associated with AMI and pneumonia in the US, 1999 to 2023. * Indicates that the AAPC is significant. The shaded area represents the 95% CI. AAMR, age-adjusted mortality rate; AMI, acute myocardial infarction; AAPC, average annual percent changes; NH, non-Hispanic; CI, confidence interval.

### 3.4 Trends by urbanization

There were 125,686 deaths (76.79%) in metropolitan, which is much higher than that of non-metropolitan with 37,996 deaths (23.21%). On the contrary, non-metropolitan had higher AAMR per 100,000 than metropolitan (22.93, 95% CI 22.69 to 23.16 vs. 16.59, 95% CI 16.5 to 16.69)(Table 1; Supplementary Table S4).

The AAMR decreased significantly for metropolitan during 1999-2018 (-5.59, 95% CI -6.07 to -5.11, *p* < 0.001) and non-metropolitan during 2002-2018 (-6.46, 95% CI −8.16 to -4.73, *p* < 0.001; -2.66, 95% CI -3.94 to -1.37, *p* < 0.001). Besides, the upward trend during 2018-2020 only significant in non-metropolitan (APC: 14.46, 95% CI 2.01 to 28.43, *p* = 0.026). In general, the metropolitan had a steeper decline than non-metropolitan (AAPC: -3.51, 95% CI -5.10 to -1.89, *p* < 0.001 vs. -1.69, 95% CI -3.04 to -0.31, *p* = 0.016)(Figure 3; Supplementary Figure S3).

**Figure 3:**
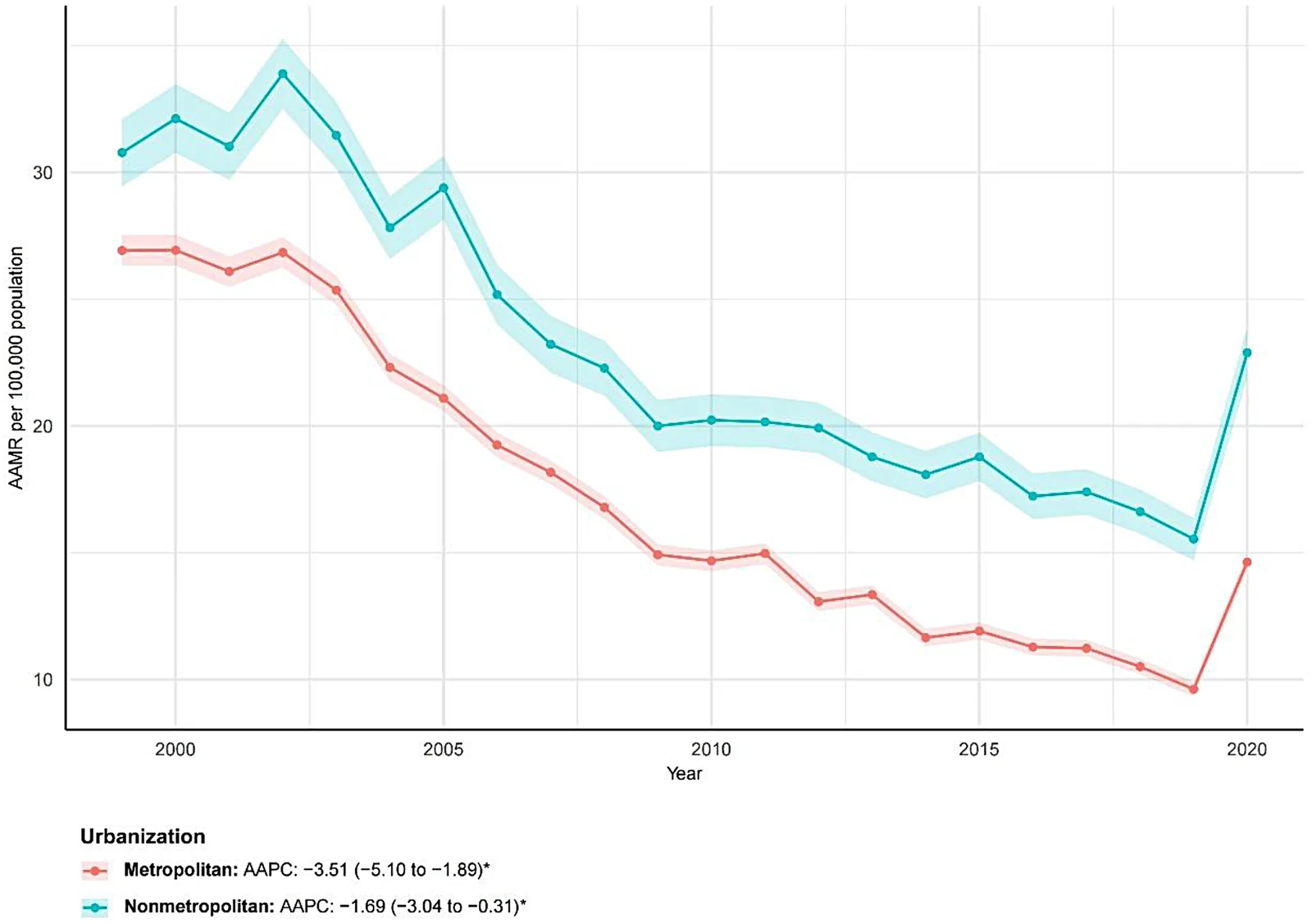
Urbanization-stratified AAMRs per 100,000 associated with AMI and pneumonia in the US, 1999 to 2020. * Indicates that the AAPC is significant. The shaded area represents the 95% CI. AAMR, age-adjusted mortality rate; AMI, acute myocardial infarction; AAPC, average annual percent changes; CI, confidence interval.

### 3.5 Trends by census region

Most of the death occurred in South region (37.25%), and this region consistently showed highest AAMR per 100,000 whether during 1999-2020 (18.14, 95% CI 17.99 to 18.29) or 2021-2023 (16.31, 95% CI 15.99 to 16.64)(Table 1; Supplementary Table S5).

All of the census region showed similar trends of AAMRs, with significant decrease before 2018, and a even steeper decline during 2021-2023. Notably, significant increase of AAMR was only identified in Midwest region (APC: 17.15, 95% CI 7.16 to 28.08, *p* = 0.002) and South region (APC: 23.80, 95% CI 2.53 to 49.49, *p* = 0.029). Totally, Northeast region demonstrated lowest AAPC (-5.29, 95% CI -8.03 to -2.47, *p* < 0.001), followed by Midwest region (-4.26, 95% CI -5.56 to -2.95, *p* < 0.001), West region (-4.01, 95% CI -6.98 to -0.95, *p* = 0.011) and South region (-3.85, 95% CI -6.37 to -1.25, *p* = 0.004)(Figure 4; Supplementary Figure S4).

**Figure 4:**
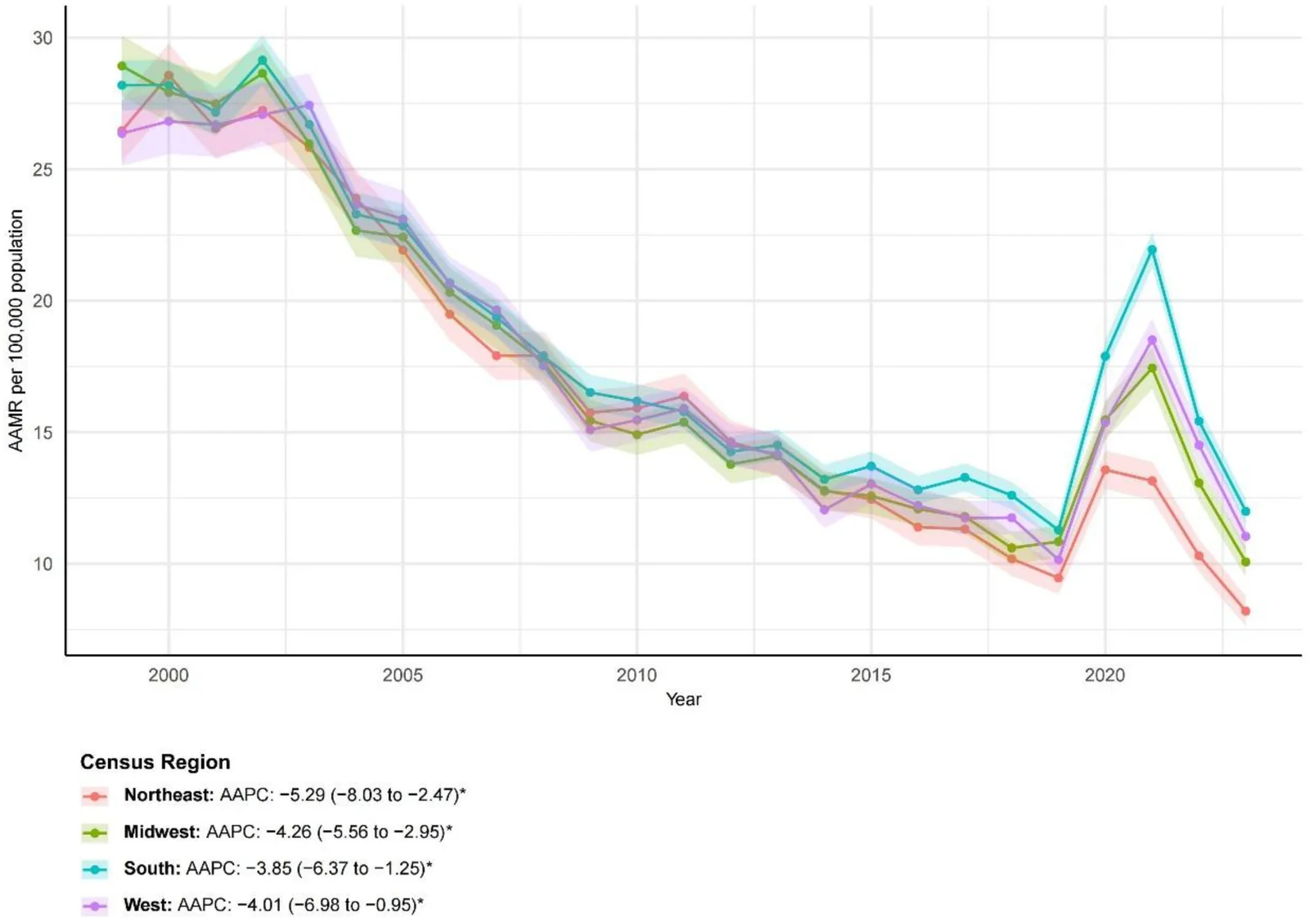
Census region-stratified AAMRs per 100,000 associated with AMI and pneumonia in the US, 1999 to 2023. * Indicates that the AAPC is significant. The shaded area represents the 95% CI. AAMR, age-adjusted mortality rate; AMI, acute myocardial infarction; AAPC, average annual percent changes; CI, confidence interval.

### 3.6 Trends by state

Between 1999 and 2020, West Virginia (31.65, 95% CI 30.28 to 33.02), Rhode Island (30.04, 95% CI 28.3 to 31.79), Kentucky (26.76, 95% CI 25.86 to 27.67) demonstrated the highest AAMR per 100,000, while the lowest were found in Utah (11.16, 95% CI 10.27 to 12.05), Arizona (10.72, 95% CI 10.26 to 11.18), Montana (10.31, 95% CI 9.21 to 11.41). Among 2021-2023, West Virginia (33.34, 95% CI 29.78 to 36.9) showed the greatest AAMR, followed by Kentucky (29.23, 95% CI 26.91 to 31.56) and District of Columbia (23.67, 95% CI 18.06 to 30.47). The top three states with lowest AAMR were Delaware (8.89, 95% CI 6.55 to 11.79), New York (8.53, 95% CI 7.96 to 9.1), and Connecticut (8.22, 95% CI 6.93 to 9.52)(Figures 5, 6; Supplementary Table S6).

**Figure 5:**
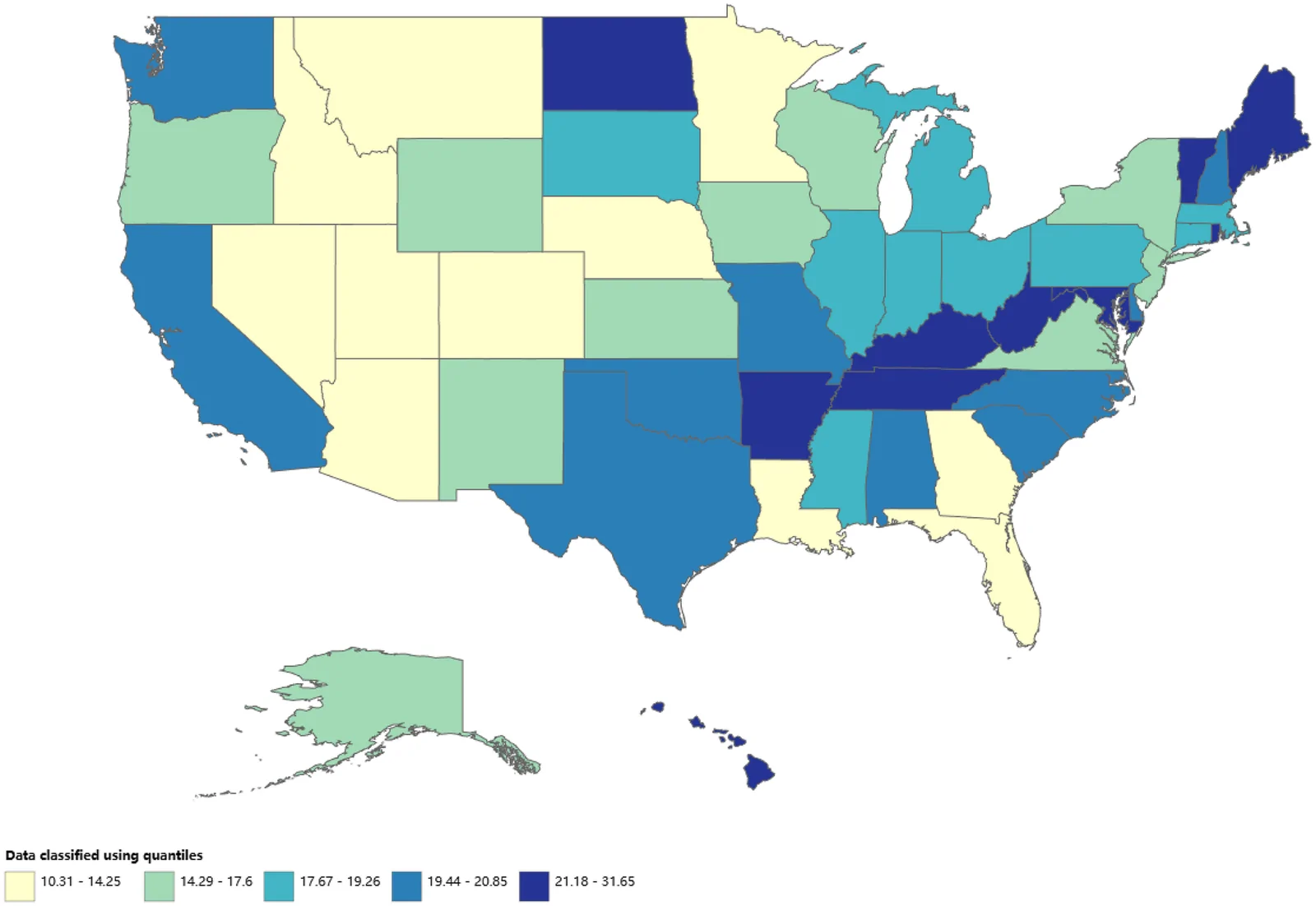
AAMRs per 100,000 associated with AMI and pneumonia stratified by state in the US, 1999 to 2020. AAMR, age-adjusted mortality rate; AMI, acute myocardial infarction.

**Figure 6:**
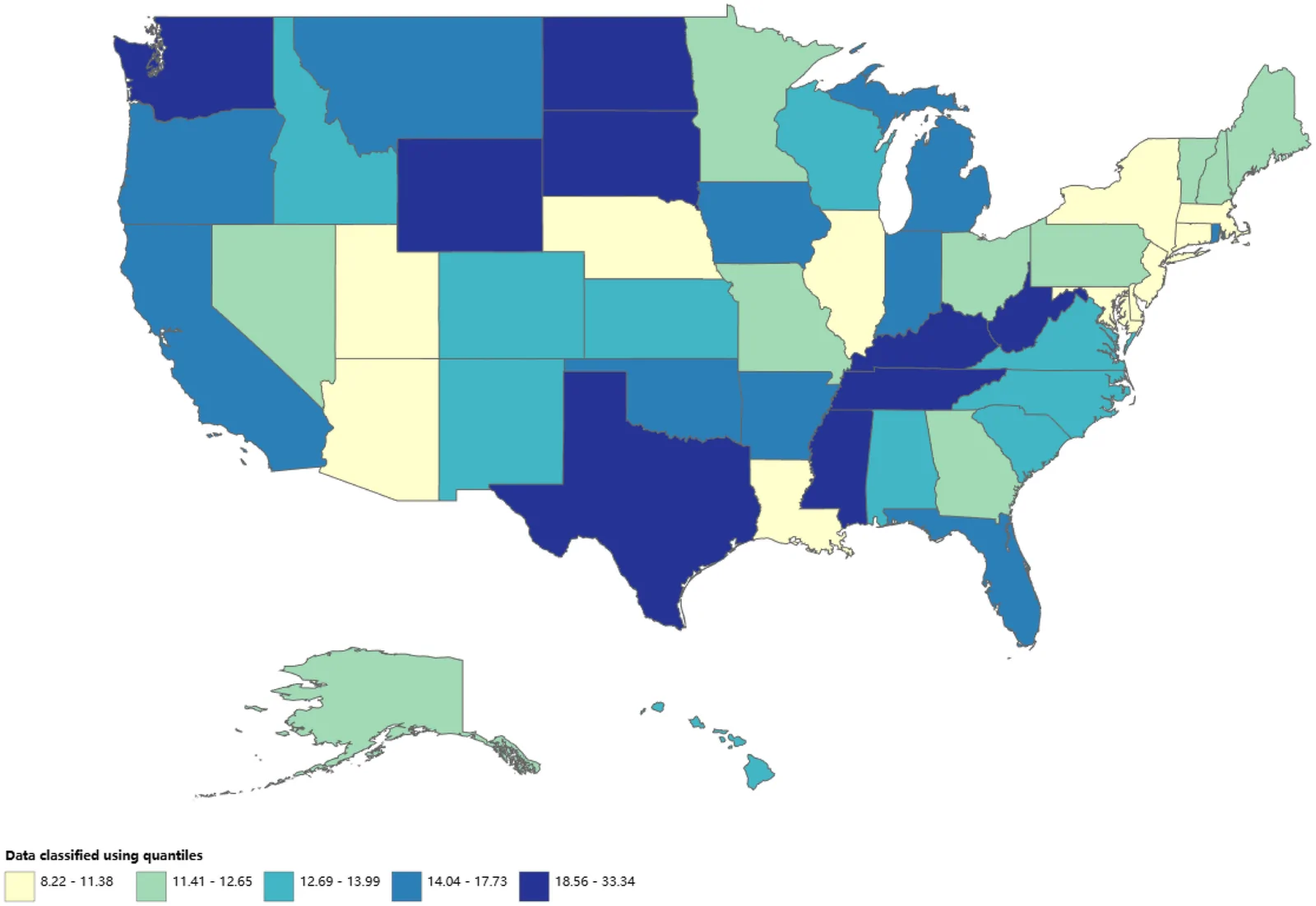
AAMRs per 100,000 associated with AMI and pneumonia stratified by state in the US, 2021 to 2023. AAMR, age-adjusted mortality rate; AMI, acute myocardial infarction.

### 3.7 Trends by ten-year age group

Older adults aged 85 + years had the highest value in death count (44.5%) and crude mortality rate (CMR) per 100,000 during 1999-2020 (62.54, 95% CI 62.09 to 62.99) and 2021-2023 (44.05, 95% CI 43.1 to 45.01) compared to other age groups (Table 1; Supplementary Table S7).

All of the age groups showed significant decreases at a part of 1999-2018 period and demonstrated sharp declines during the full period of 2021-2023. Between 2018 and 2021, rapid increase of CMR occurred among elderly aged 65-84 years. Overall, older adults aged 75-84 years demonstrated the steepest decline (AAPC: -4.14, 95% CI -5.49 to -2.77, *p* < 0.001) compared to age group of 65-74 years (AAPC: -3.89, 95% CI -6.23 to -1.50, *p* = 0.002) and 85 + years (AAPC: -3.86, 95% CI -6.09 to -1.58, *p* = 0.001)(Figure 7; Supplementary Figure S5).

**Figure 7:**
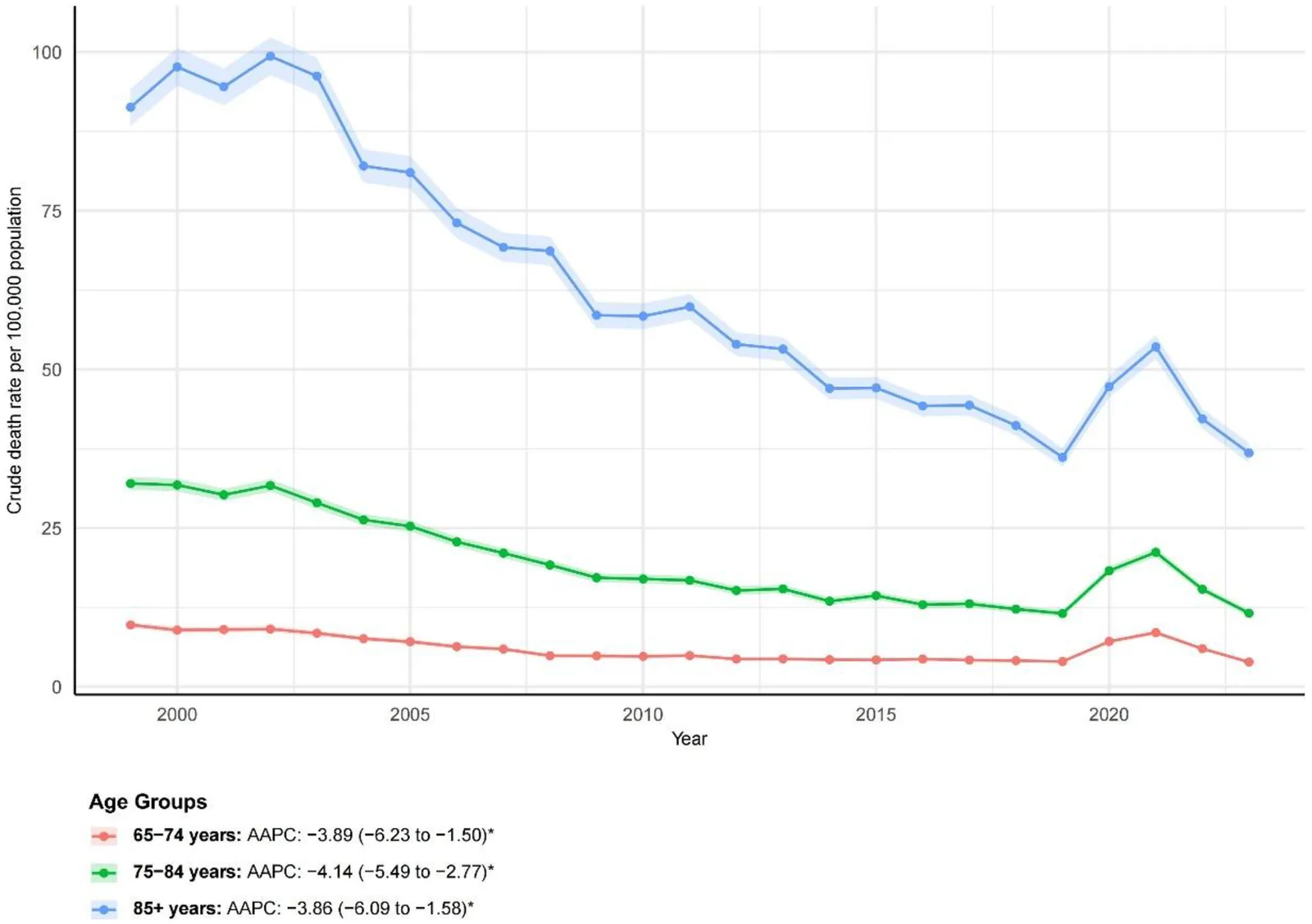
Age-stratified crude mortality rates per 100,000 associated with AMI and pneumonia in the US, 1999 to 2023. * Indicates that the AAPC is significant. The shaded area represents the 95% CI. AMI, acute myocardial infarction; AAPC, average annual percent changes; CI, confidence interval.

### 3.8 Trends by place of death

In total, there were 186,021 death certificates available for place of death analysis. Hospice-facility data were unavailable for 1999–2002, and the 2003 count was suppressed by CDC WONDER because it involved fewer than 10 deaths. The suppressed count was treated as missing rather than zero. Most of the death was identified in Medical Facility (82.65%), Nursing Home/Long Term Care (10.06%), and Decedent's Home (4.05%) (Table 1; Supplementary Table S8).

### 3.9 Sensitivity analysis

A total of 79,980 deaths related to AMI and pneumonia were identified during 1999-2023 in sensitivity analysis. The AAMR per 100,000 was 7.95 (95% CI 7.89 to 8.00) during 1999-2020 and 4.16 (95% CI 4.05 to 4.26) between 2021 and 2023. The AAMR decreased insignificantly in 1999-2002 period (APC: -1.96, 95% CI -5.91 to 2.16, *p* = 0.325), and significantly declined afterwards, with a rapid decrease between 2002 and 2009 (APC: -8.71, 95% CI -10.10 to -7.29, *p* < 0.001), and a slight decline during 2009-2023 (APC: -4.44, 95% CI -4.94 to -3.93, *p* < 0.001), which revealed a downward trend in general (AAPC: -5.40, 95% CI -6.05 to -4.74, *p* < 0.001). The outcome of sensitivity analysis was mostly consistent with the primary analysis (Supplementary Table S9; Supplementary Figures S6, S7).

## 4 Discussion

To the best of our knowledge, this is the first study assessing the mortality trends of older patients with AMI and pneumonia during 1999-2023 based on the CDC wonder database. The significant decline of AAMR was observed in overall population and each subgroups through the full study period. Men, NH American Indian or Alaska Native, rural area, South region, elderly aged 85 + years were high-risk subgroup, which showed the highest AAMR/CMR and AAPC compared to other subgroups between 1999 and 2023. Notably, there was a significant increase of AAMR for men, NH Black, Midwest region, South region, individuals aged 65–84 years during 2018-2021, Hispanic during 2017-2021, non-metropolitan during 2018-2020. These populations warrant additional attention of public health policies and health professionals for targeted screening and preventive strategies against AMI and pneumonia.

The overall trends in mortality associated with AMI and pneumonia showed a significant decline (AAPC:-4.26, 95% CI -6.71 to -1.74), although an insignificant reverse pattern occurred during COVID-19 Pandemic from 2018 to 2021. Previous study showed that there was a decline in AMI-related AAMR with an AAPC of −4.96 (95% CI −5.11 to −4.81) during 1999-2019 (22). Meanwhile, another study using CDC wonder database indicated that the AAMR related to pneumonia decreased between 1999 and 2022 in the US with AAPC of -3.1 (95% CI −3.6 to −2.6) (11). Several factors may have contributed to the decline in mortality rates, including the implementation of prehospital EKGs by emergency medical services (EMS), statewide adoption of STEMI hospital destination protocols that allow EMS to bypass non-PCI-capable hospitals, faster time to reperfusion through PCI, and the more widespread use of primary PCI medications (23, 24). Additionally, the broader application of guideline-directed therapies, improved management of chronic comorbidities, and enhanced post-AMI care and revascularization practices may also have contributed to the overall decline in AMI mortality. Additionally, from 2005 to 2014, the CDC reported a significant increase in the number of individuals receiving the influenza vaccine, with coverage doubling to 43.7%. Vaccine coverage is highest among the elderly population, and 58.7% of older adults reported receiving at least one type of pneumococcal vaccine in 2014 (25). The proportion of adults aged ≥65 years who received at least one pneumococcal vaccine increased from 41% to 63% between 2010 and 2019, and the vaccination rates for any pneumococcal vaccine rose from 49.6% in the 65–69 age group to 75.3% in those aged >85 years (26).

Men with AMI and pneumonia had higher AAMR and lower AAMR reduction than women. Consistently, the AAMR and AAPC of AMI-associated death were higher in men compared to women from 1999 to 2019 (22). For death associated with pneumonia, despite higher overall AAMR in men, both sexes showed similar AAPC between 1999 and 2022 (11). Men consistently exhibited a higher AAMR each year, which may be partly related to hormonal differences that provide a protective effect for women. This well-documented advantage slows the progression of atherosclerotic cardiovascular disease (ASCVD) in women compared to men (27). Furthermore, men tend to have a higher prevalence of traditional risk factors, including dyslipidemia, hypertension, smoking, and alcohol use, all of which contribute to an increased incidence of AMI (28, 29). In addition, women are more likely to receive influenza vaccination compared to men (25). Previous studies also indicated that the higher burden of severe pneumonia in men may due to male-specific environmental factors (such as higher rates of smoking) and immunologic factors (30–32).

NH American Indian or Alaska Native showed the highest AAMR and AAPC compared to other races. However, NH Black or African American had the highest AAMR of AMI or pneumonia in the US (11, 22). This may due to social determinants of health, including limited access to healthcare or insurance, higher prevalence of comorbidities such as diabetes and obesity, as well as reduced rates of revascularization in AMI cases, insufficient prevention efforts, and an increased burden of comorbid conditions (33–38).

Non-metropolitan died from AMI and pneumonia demonstrated higher AAMR and slighter AAMR reduction than metropolitan, which is consistent with previous studies focusing on death related to AMI or pneumonia alone (11, 22). Lower mortality rates in urban areas may be partly related to faster arrival to emergency department and quicker transfer to hospitals equipped for PCI/CABG procedures, resulting in higher rates of interventional treatments (39). In comparisons between rural and urban areas, the rate of cardiac catheterization within 30 days of AMI is 49.7% vs. 63.6%, while PCI rates are 42.1% versus 45.7% (40). Non-metropolitan experience prolonged intervals from 911 activation to hospital arrival and, when interfacility transfer is required, substantially longer transport times, largely because advanced cardiac services are far less available than in urban settings (39, 41). In contrast, greater access to ASCVD preventive care in urban areas may help reduce the prevalence of smoking, obesity, and hypertension, which in turn is associated with persistently lower AMI incidence and mortality in these populations (42).

South region demonstrated the highest AAMR and AAPC compared to other census regions, which is differ from previous researches. The region with the highest AAPC of AAMR related to AMI or pneumonia was West region during 1999-2019 and Northeast region and Midwest region during 1999-2022, respectively (11, 22). Cardiovascular risk factors such as obesity, hypertension, smoking, and diabetes continue to be more prevalent in the South, largely driven by poverty and limited awareness, which are exacerbated by lower levels of healthcare literacy and access. These factors may partly explain the higher AMI mortality reported in this region (43).

Notably, West Virginia and Kentucky are listed as top three states with highest AAMR during whether 1999-2020 or 2021-2023. This may be due to the socio-economic backwardness and lack of health resources in these states, high burdens of chronic diseases and health behavior risks, environmental/occupational exposure, and delays in medical prevention and acute care.

The risk of death is relatively high among older adults aged 85 + years. As age increases, the burden of morbidity also rises, thereby elevating the risk of mortality from AMI. Additionally, older adults are more likely to have contraindications that prevent them from being eligible for reperfusion therapy, and even when they do qualify, they are less likely to receive it (44). The presentation of AMI in elderly patients is further complicated by the tendency to exhibit atypical symptoms, which can delay the prompt recognition of the condition and, consequently, hinder timely treatment (45).

The significant increase of AAMR in men,NH Black, Hispanic, non-metropolitan, Midwest region, South region, individuals aged 65–84 years, from 2017/2018 to 2020/2021 may have been temporally associated with the COVID-19 Pandemic. Global data indicates that the highest number of cardiovascular-related deaths occurred in 2020, coinciding with the first year of the pandemic, marking a significant peak since 2003 (46). One study reported a 43% decrease in urgent cardiac admissions in March 2020, along with an increase in mortality rates compared to previous years (47). Similarly, it was reported that the AMI-associated AAMR increase during the pandemic was higher among male, Hispanic, South region, and rural area (22). Besides, patients aged <45 years had the highest increase of AAMR related to cardiovascular disease during the pandemic with a percent change of 14.16%, followed by age group of 45–64 years (10.53%), and ≥65 years (4.77%) (48). Several COVID-19-related factors, including infection and delayed medical care, may have contributed to the observed increase in AAMR. The pandemic significantly disrupted healthcare systems, leading to overcrowded emergency departments, and many individuals opted to avoid medical facilities to minimize exposure to the virus. Financial concerns also played a role, resulting in postponed presentations and delayed interventions for such disease (49). Moreover, patients may have been less likely to attend outpatient appointments, hindering effective monitoring of risk factors. The pandemic also brought about lifestyle changes, including an increase in sedentary behavior and a shift to working from home, both of which likely had a lasting negative impact on cardiovascular health. Additionally, COVID-19 infection has been shown to trigger systemic inflammation, which leads to endothelial activation, hypercoagulability, and thrombosis in both arteries and veins, which may potentially contribute to increased AMI mortality (50, 51).

A higher proportion of deaths occurred in Medical Facility (82.65%) than in other places. This may indicate that the majority of decedents have severe conditions, requiring continuous monitoring and treatment in a medical facility.

Our findings highlight substantial disparities in outcomes among specific subgroups of patients, underscoring the need for focused interventions. Public health strategies tailored to high-risk populations—particularly men, NH American Indian or Alaska Native, rural area, South region, elderly aged 85 + years—will be essential to lowering mortality from AMI and pneumonia. Promoting vaccination programs and cardiovascular health education through policy measures could further lessen the burden of AMI and pneumonia in these vulnerable groups. Policy reforms are also needed to support equity for accessing therapeutic services in resource-limited areas. Multidisciplinary teams should be involved in the therapeutic trajectory to mitigate socioeconomic and practical obstacles, which hinder access to preventive and therapeutic interventions.

This study has several limitations result from using CDC wonder database: First, dependence on death certificate records and ICD-10 coding may fail to fully reflect the true burden of AMI and pneumonia as the cause of death, considering that misclassification may occur due to miscoding and misdiagnosis. Second, the database does not contain granular clinical data, such as disease severity, comorbidity profiles, or specific treatment regimens, which restricts our ability to evaluate the effects of pharmacologic therapies, trajectories of disease progression, and the role of multimorbidity in shaping mortality patterns. The lack of baseline sociodemographic variables, including socioeconomic status and healthcare access, further limits a thorough assessment of inequities in care delivery and outcomes. Third, the unavailable and suppressed data may have limited the comprehensiveness and accuracy of subgroup analysis. Moreover, interstate migration is not accounted for in the database, which may compromise the precision of state-level mortality estimates. Finally, racial classification on death certificates is derived from information supplied by the individual completing the form and may, in some instances, rely on assumptions when direct confirmation is not available, thereby introducing potential misclassification bias in the reported racial and ethnic data.

## 5 Conclusions

There is a decline in mortality rate associated with pneumonia and AMI among older adults in the US during 1999-2023, which is consistent with all subgroups. Meanwhile, men, NH American Indian or Alaska Native, non-metropolitan, living in South region, and elderly aged 85 + years were high-risk population. These results underscore the urgent need for comprehensive public health approaches that prioritize expanded screening efforts, improved access to evidence-based treatments, and tailored interventions for populations at highest risk. Sustained surveillance and ongoing evaluation of intervention effectiveness will be essential to mitigate this public health crisis.

## Data Availability

Publicly available datasets were analyzed in this study. This data can be found here: CDC wonder database (https://wonder.cdc.gov/).
