# Supplementary material for: Trends in mortality due to acute myocardial infarction and pneumonia among older adults in the U.S.: a CDC wonder analysis (1999-2023)
Source: Front Cardiovasc Med. 2026 Sep 30;13:1913867. doi: 10.3389/fcvm.2026.1913867 (PMC13661469; doi:10.3389/fcvm.2026.1913867)
Supplement: Supplementary file 1 [file Datasheet1.docx]

Supplemental materials

Supplemental Table 1: Deaths due to AMI and pneumonia, stratified by sex and race, in adults of age ≥65 in the United States, 1999 to 2023

| Deaths | | | | | | | | | |
| --- | --- | --- | --- | --- | --- | --- | --- | --- | --- |
| Year | Overall | Female | Male | NH White | NH Black or African American | NH Asian or Pacific Islander | NH American Indian or Alaska Native | Hispanic or Latino | Population |
| 1999 | 9503 | 4582 | 4921 | 8221 | 709 | 178 | 25 | 343 | 34797841 |
| 2000 | 9713 | 4707 | 5006 | 8346 | 748 | 179 | 32 | 386 | 34991753 |
| 2001 | 9541 | 4562 | 4979 | 8159 | 696 | 216 | 31 | 414 | 35290291 |
| 2002 | 10059 | 4807 | 5252 | 8607 | 762 | 234 | 31 | 399 | 35522207 |
| 2003 | 9594 | 4610 | 4984 | 8226 | 681 | 212 | 56 | 397 | 35863529 |
| 2004 | 8561 | 4064 | 4497 | 7272 | 647 | 210 | 38 | 374 | 36203319 |
| 2005 | 8450 | 4182 | 4268 | 7163 | 621 | 226 | 34 | 394 | 36649798 |
| 2006 | 7760 | 3883 | 3877 | 6579 | 551 | 222 | 45 | 351 | 37164107 |
| 2007 | 7416 | 3559 | 3857 | 6258 | 541 | 234 | 30 | 345 | 37825711 |
| 2008 | 7079 | 3545 | 3534 | 5984 | 492 | 213 | 27 | 353 | 38777621 |
| 2009 | 6412 | 3173 | 3239 | 5376 | 464 | 180 | 33 | 351 | 39623175 |
| 2010 | 6467 | 3069 | 3398 | 5426 | 453 | 210 | 23 | 344 | 40267984 |
| 2011 | 6750 | 3249 | 3501 | 5616 | 465 | 243 | 40 | 375 | 41394141 |
| 2012 | 6240 | 2960 | 3280 | 5105 | 473 | 246 | 30 | 377 | 43145356 |
| 2013 | 6395 | 3055 | 3340 | 5252 | 482 | 239 | 31 | 377 | 44704074 |
| 2014 | 5867 | 2825 | 3042 | 4794 | 425 | 214 | 36 | 378 | 46243211 |
| 2015 | 6127 | 2939 | 3188 | 4988 | 465 | 216 | 32 | 404 | 47760852 |
| 2016 | 5912 | 2731 | 3181 | 4841 | 424 | 227 | 29 | 383 | 49244195 |
| 2017 | 6041 | 2747 | 3294 | 4849 | 467 | 242 | 41 | 429 | 50858679 |
| 2018 | 5831 | 2658 | 3173 | 4597 | 445 | 259 | 39 | 475 | 52431193 |
| 2019 | 5483 | 2418 | 3065 | 4311 | 484 | 215 | 34 | 426 | 54058263 |
| 2020 | 8481 | 3481 | 5000 | 6093 | 815 | 387 | 77 | 1090 | 55659365 |
| 2021 | 9508 | 3844 | 5664 | 7121 | 861 | 430 | 55 | 988 | 55847953 |
| 2022 | 7455 | 3071 | 4384 | 5672 | 642 | 339 | 65 | 690 | 57794852 |
| 2023 | 5766 | 2455 | 3311 | 4446 | 466 | 261 | 36 | 518 | 59248361 |
| Total | 186411 | 87176 | 99235 | 153302 | 14279 | 6032 | 950 | 11361 | 1101367831 |

Supplemental Table 2: Overall and sex-stratified age-adjusted mortality rates per 100,000 associated with AMI and pneumonia in adults aged ≥65 in the United States, 1999 to 2023

| AAMR (95% CI) | | | |
| --- | --- | --- | --- |
| Year | Overall | Female | Male |
| 1999 | 27.63 (27.07-28.18) | 20.84 (20.24-21.45) | 39.93 (38.79-41.07) |
| 2000 | 27.92 (27.37-28.48) | 20.97 (20.37-21.57) | 40.1 (38.97-41.23) |
| 2001 | 27.01 (26.47-27.56) | 20.17 (19.58-20.76) | 39.23 (38.12-40.33) |
| 2002 | 28.19 (27.64-28.74) | 21.07 (20.47-21.67) | 40.66 (39.54-41.78) |
| 2003 | 26.48 (25.95-27.01) | 19.94 (19.36-20.52) | 37.87 (36.8-38.94) |
| 2004 | 23.38 (22.88-23.87) | 17.48 (16.94-18.02) | 33.38 (32.39-34.36) |
| 2005 | 22.63 (22.14-23.11) | 17.8 (17.26-18.34) | 31.1 (30.15-32.04) |
| 2006 | 20.35 (19.9-20.8) | 16.16 (15.65-16.68) | 27.37 (26.5-28.24) |
| 2007 | 19.06 (18.63-19.49) | 14.54 (14.06-15.03) | 26.53 (25.68-27.37) |
| 2008 | 17.8 (17.39-18.22) | 14.17 (13.7-14.65) | 23.83 (23.04-24.62) |
| 2009 | 15.84 (15.45-16.23) | 12.52 (12.08-12.97) | 21.15 (20.42-21.88) |
| 2010 | 15.71 (15.32-16.09) | 11.96 (11.53-12.39) | 21.76 (21.03-22.5) |
| 2011 | 15.87 (15.49-16.25) | 12.32 (11.89-12.75) | 21.6 (20.89-22.32) |
| 2012 | 14.32 (13.96-14.68) | 10.96 (10.56-11.36) | 19.53 (18.85-20.2) |
| 2013 | 14.29 (13.94-14.64) | 11.16 (10.75-11.56) | 19.14 (18.49-19.8) |
| 2014 | 12.8 (12.47-13.13) | 10.13 (9.75-10.51) | 16.81 (16.2-17.41) |
| 2015 | 13.08 (12.75-13.41) | 10.35 (9.97-10.73) | 17.15 (16.55-17.75) |
| 2016 | 12.3 (11.98-12.62) | 9.42 (9.06-9.77) | 16.5 (15.92-17.08) |
| 2017 | 12.29 (11.98-12.6) | 9.31 (8.96-9.66) | 16.58 (16.01-17.15) |
| 2018 | 11.51 (11.22-11.81) | 8.79 (8.46-9.13) | 15.48 (14.93-16.02) |
| 2019 | 10.6 (10.32-10.88) | 7.87 (7.55-8.19) | 14.47 (13.96-14.99) |
| 2020 | 16.01 (15.66-16.35) | 11.24 (10.87-11.62) | 22.59 (21.96-23.23) |
| Total (1999-2020) | 17.76 (17.67-17.85) | 13.64 (13.54-13.73) | 24.18 (24.02-24.34) |
| 2021 | 18.53 (18.15-18.9) | 13.04 (12.63-13.46) | 26.16 (25.47-26.86) |
| 2022 | 13.78 (13.46-14.09) | 9.7 (9.36-10.05) | 19.6 (19.01-20.19) |
| 2023 | 10.67 (10.39-10.95) | 7.86 (7.55-8.17) | 14.6 (14.1-15.11) |
| Total (2021-2023) | 14.2 (14.02-14.39) | 10.13 (9.93-10.34) | 19.99 (19.64-20.33) |

Supplemental Table 3: Race-stratified age-adjusted mortality rates per 100,000 associated with AMI and pneumonia in adults aged ≥65 in the United States, 1999 to 2023

| AAMR (95% CI) | | | | | |
| --- | --- | --- | --- | --- | --- |
| Year | NH White | NH American Indian or Alaska Native | Hispanic or Latino | NH Black or African American | NH Asian or Pacific Islander |
| 1999 | 27.78 (27.18-28.38) | 23.55 (15.09-35.04) | 25.09 (22.39-27.8) | 26.69 (24.72-28.67) | 28.78 (24.45-33.12) |
| 2000 | 27.93 (27.33-28.53) | 25.97 (17.65-36.86) | 27.07 (24.33-29.81) | 28.04 (26.03-30.06) | 26.36 (22.41-30.31) |
| 2001 | 27.02 (26.43-27.61) | 24.42 (16.47-34.86) | 27.16 (24.5-29.82) | 25.86 (23.93-27.79) | 29.19 (25.21-33.16) |
| 2002 | 28.26 (27.66-28.85) | 24.79 (16.73-35.39) | 24.91 (22.42-27.39) | 28.05 (26.05-30.05) | 29.64 (25.78-33.5) |
| 2003 | 26.67 (26.09-27.24) | 42.47 (31.91-55.42) | 23.54 (21.19-25.9) | 24.75 (22.88-26.61) | 25.71 (22.2-29.23) |
| 2004 | 23.4 (22.86-23.93) | 27.93 (19.56-38.67) | 20.98 (18.82-23.14) | 23.37 (21.56-25.18) | 23.16 (19.98-26.34) |
| 2005 | 22.69 (22.17-23.22) | 24.76 (16.94-34.96) | 20.5 (18.44-22.55) | 21.91 (20.18-23.64) | 23.36 (20.27-26.44) |
| 2006 | 20.51 (20.01-21.01) | 32.79 (23.83-44.02) | 17.86 (15.97-19.75) | 18.94 (17.35-20.53) | 22.15 (19.21-25.09) |
| 2007 | 19.19 (18.71-19.67) | 20.97 (14.04-30.11) | 16.57 (14.81-18.34) | 18.39 (16.83-19.95) | 21.85 (19.03-24.67) |
| 2008 | 17.96 (17.5-18.42) | 17.06 (11.14-25) | 16.18 (14.48-17.88) | 16.28 (14.84-17.73) | 18.84 (16.29-21.38) |
| 2009 | 15.94 (15.51-16.37) | 21.66 (14.81-30.57) | 14.73 (13.18-16.29) | 14.85 (13.5-16.21) | 14.76 (12.59-16.93) |
| 2010 | 15.85 (15.43-16.27) | 13.52 (8.47-20.47) | 14.06 (12.57-15.56) | 14.1 (12.79-15.4) | 16.7 (14.43-18.97) |
| 2011 | 16.03 (15.6-16.45) | 21.91 (15.5-30.07) | 13.98 (12.55-15.4) | 14.04 (12.75-15.32) | 17.45 (15.24-19.65) |
| 2012 | 14.25 (13.86-14.64) | 15.41 (10.24-22.27) | 13.33 (11.98-14.69) | 13.68 (12.44-14.92) | 16.56 (14.48-18.64) |
| 2013 | 14.47 (14.07-14.86) | 15.18 (10.17-21.8) | 12.52 (11.25-13.79) | 13.38 (12.18-14.59) | 14.59 (12.73-16.45) |
| 2014 | 12.95 (12.58-13.32) | 16.95 (11.74-23.69) | 11.66 (10.48-12.85) | 11.37 (10.28-12.46) | 12.14 (10.5-13.77) |
| 2015 | 13.34 (12.97-13.72) | 14.1 (9.58-20.02) | 11.72 (10.57-12.88) | 12.02 (10.92-13.13) | 11.43 (9.9-12.97) |
| 2016 | 12.67 (12.31-13.04) | 11.33 (7.47-16.49) | 10.53 (9.46-11.59) | 10.48 (9.47-11.5) | 11.18 (9.71-12.64) |
| 2017 | 12.46 (12.11-12.81) | 15.18 (10.8-20.76) | 11.15 (10.09-12.22) | 11.08 (10.06-12.1) | 11.21 (9.78-12.63) |
| 2018 | 11.6 (11.26-11.93) | 14.25 (10.03-19.64) | 11.63 (10.57-12.69) | 10.25 (9.28-11.22) | 11.37 (9.98-12.77) |
| 2019 | 10.71 (10.39-11.03) | 11.79 (8.11-16.55) | 10.11 (9.14-11.08) | 10.65 (9.68-11.61) | 8.84 (7.65-10.03) |
| 2020 | 14.88 (14.51-15.26) | 25.62 (20.12-32.17) | 24.16 (22.71-25.61) | 17.19 (15.99-18.39) | 14.83 (13.34-16.32) |
| Total (1999-2020) | 18.01 (17.91-18.11) | 19.67 (18.28-21.07) | 15.78 (15.45-16.11) | 16.63 (16.34-16.93) | 16.15 (15.7-16.6) |
| 2021 | 18.28 (17.85-18.71) | 17.7 (13.22-23.21) | 21.97 (20.58-23.36) | 18.51 (17.24-19.78) | 16.9 (15.29-18.52) |
| 2022 | 13.77 (13.41-14.13) | 20.93 (16.08-26.78) | 14.61 (13.51-15.71) | 13.3 (12.25-14.36) | 12.48 (11.14-13.81) |
| 2023 | 10.9 (10.58-11.22) | 11.11 (7.74-15.45) | 10.73 (9.79-11.66) | 9.58 (8.69-10.47) | 9.32 (8.18-10.45) |
| Total (2021-2023) | 14.25 (14.04-14.47) | 16.53 (13.88-19.17) | 15.56 (14.9-16.22) | 13.68 (13.06-14.3) | 12.75 (11.96-13.53) |

Supplemental Table 4: Urbanization-stratified age-adjusted mortality rates per 100,000 associated with AMI and pneumonia in adults aged ≥65 in the United States, 1999 to 2020

| AAMR (95% CI) | | |
| --- | --- | --- |
| Year | Metropolitan | Nonmetropolitan |
| 1999 | 26.92 (26.31-27.54) | 30.78 (29.45-32.1) |
| 2000 | 26.93 (26.32-27.54) | 32.12 (30.77-33.48) |
| 2001 | 26.09 (25.49-26.68) | 31.02 (29.7-32.35) |
| 2002 | 26.84 (26.25-27.44) | 33.89 (32.5-35.27) |
| 2003 | 25.35 (24.77-25.92) | 31.46 (30.13-32.78) |
| 2004 | 22.31 (21.78-22.85) | 27.82 (26.58-29.07) |
| 2005 | 21.09 (20.58-21.61) | 29.39 (28.12-30.66) |
| 2006 | 19.25 (18.76-19.74) | 25.18 (24.01-26.36) |
| 2007 | 18.17 (17.7-18.64) | 23.22 (22.1-24.34) |
| 2008 | 16.79 (16.34-17.23) | 22.28 (21.19-23.36) |
| 2009 | 14.92 (14.5-15.33) | 20 (18.97-21.03) |
| 2010 | 14.68 (14.27-15.09) | 20.23 (19.2-21.25) |
| 2011 | 14.97 (14.56-15.37) | 20.16 (19.15-21.17) |
| 2012 | 13.07 (12.69-13.45) | 19.92 (18.92-20.92) |
| 2013 | 13.35 (12.97-13.72) | 18.78 (17.82-19.75) |
| 2014 | 11.65 (11.31-12) | 18.08 (17.14-19.02) |
| 2015 | 11.91 (11.57-12.26) | 18.78 (17.83-19.73) |
| 2016 | 11.28 (10.95-11.61) | 17.23 (16.33-18.13) |
| 2017 | 11.23 (10.9-11.56) | 17.4 (16.5-18.3) |
| 2018 | 10.51 (10.2-10.82) | 16.62 (15.75-17.49) |
| 2019 | 9.62 (9.32-9.92) | 15.54 (14.7-16.37) |
| 2020 | 14.63 (14.27-14.99) | 22.89 (21.89-23.89) |
| Total | 16.59 (16.5-16.69) | 22.93 (22.69-23.16) |

Supplemental Table 5: Census region-stratified age-adjusted mortality rates per 100,000 associated with AMI and pneumonia in adults aged ≥65 in the United States, 1999 to 2023

| AAMR (95% CI) | | | | |
| --- | --- | --- | --- | --- |
| Year | Northeast | Midwest | South | West |
| 1999 | 26.46 (25.29-27.63) | 28.92 (27.77-30.07) | 28.19 (27.24-29.14) | 26.36 (25.13-27.6) |
| 2000 | 28.57 (27.36-29.78) | 27.92 (26.79-29.05) | 28.2 (27.25-29.15) | 26.82 (25.58-28.05) |
| 2001 | 26.54 (25.38-27.69) | 27.48 (26.37-28.6) | 27.16 (26.24-28.09) | 26.68 (25.47-27.9) |
| 2002 | 27.24 (26.07-28.4) | 28.64 (27.5-29.77) | 29.14 (28.18-30.09) | 27.07 (25.86-28.28) |
| 2003 | 25.82 (24.69-26.95) | 25.96 (24.89-27.03) | 26.7 (25.79-27.6) | 27.43 (26.22-28.64) |
| 2004 | 23.89 (22.81-24.97) | 22.67 (21.67-23.67) | 23.29 (22.45-24.13) | 23.65 (22.54-24.76) |
| 2005 | 21.92 (20.89-22.95) | 22.42 (21.43-23.4) | 22.85 (22.02-23.67) | 23.1 (22.02-24.18) |
| 2006 | 19.48 (18.51-20.44) | 20.32 (19.39-21.25) | 20.67 (19.9-21.44) | 20.64 (19.63-21.65) |
| 2007 | 17.91 (16.99-18.83) | 19.06 (18.16-19.95) | 19.37 (18.63-20.11) | 19.64 (18.67-20.61) |
| 2008 | 17.91 (16.99-18.82) | 17.66 (16.81-18.52) | 17.88 (17.18-18.59) | 17.53 (16.62-18.43) |
| 2009 | 15.74 (14.89-16.59) | 15.44 (14.65-16.24) | 16.51 (15.84-17.18) | 15.09 (14.26-15.92) |
| 2010 | 15.91 (15.05-16.76) | 14.91 (14.13-15.69) | 16.18 (15.52-16.83) | 15.46 (14.63-16.29) |
| 2011 | 16.37 (15.52-17.23) | 15.38 (14.59-16.16) | 15.78 (15.15-16.42) | 15.9 (15.07-16.73) |
| 2012 | 14.63 (13.82-15.44) | 13.78 (13.05-14.52) | 14.26 (13.67-14.86) | 14.51 (13.73-15.29) |
| 2013 | 14.1 (13.32-14.89) | 14.1 (13.36-14.83) | 14.51 (13.92-15.1) | 14.16 (13.4-14.92) |
| 2014 | 12.79 (12.05-13.54) | 12.75 (12.05-13.44) | 13.21 (12.65-13.76) | 12.05 (11.36-12.74) |
| 2015 | 12.45 (11.72-13.18) | 12.58 (11.89-13.27) | 13.71 (13.15-14.27) | 13.03 (12.32-13.74) |
| 2016 | 11.39 (10.7-12.08) | 12.08 (11.42-12.75) | 12.81 (12.28-13.34) | 12.21 (11.54-12.88) |
| 2017 | 11.32 (10.64-12) | 11.79 (11.14-12.45) | 13.28 (12.75-13.82) | 11.74 (11.09-12.39) |
| 2018 | 10.19 (9.55-10.83) | 10.6 (9.99-11.21) | 12.6 (12.08-13.11) | 11.75 (11.11-12.39) |
| 2019 | 9.46 (8.85-10.07) | 10.84 (10.23-11.45) | 11.28 (10.81-11.76) | 10.15 (9.56-10.73) |
| 2020 | 13.57 (12.84-14.3) | 15.47 (14.74-16.2) | 17.89 (17.3-18.48) | 15.37 (14.66-16.08) |
| Total (1999-2020) | 17.43 (17.24-17.62) | 17.73 (17.55-17.91) | 18.14 (17.99-18.29) | 17.3 (17.12-17.49) |
| 2021 | 13.15 (12.42-13.87) | 17.44 (16.65-18.23) | 21.94 (21.27-22.61) | 18.51 (17.71-19.31) |
| 2022 | 10.3 (9.68-10.93) | 13.07 (12.41-13.73) | 15.42 (14.88-15.96) | 14.51 (13.83-15.19) |
| 2023 | 8.2 (7.64-8.76) | 10.07 (9.49-10.66) | 11.99 (11.51-12.47) | 11.04 (10.44-11.63) |
| Total (2021-2023) | 10.47 (10.1-10.84) | 13.42 (13.02-13.81) | 16.31 (15.99-16.64) | 14.57 (14.17-14.97) |

Supplemental Table 6: State-stratified age-adjusted mortality rates per 100,000 associated with AMI and pneumonia in adults aged ≥65 in the United States

| AAMR (95% CI) | | |
| --- | --- | --- |
| State | 1999-2020 | 2021-2023 |
| Alabama | 19.74 (19-20.47) | 13.63 (12.17-15.09) |
| Alaska | 14.29 (11.9-16.67) | 12.65 (8.34-18.4) |
| Arizona | 10.72 (10.26-11.18) | 9.79 (8.8-10.78) |
| Arkansas | 24.09 (23.08-25.09) | 17.73 (15.59-19.87) |
| California | 20.04 (19.76-20.32) | 15.61 (15.02-16.21) |
| Colorado | 11.34 (10.74-11.94) | 13.12 (11.64-14.59) |
| Connecticut | 17.67 (16.93-18.41) | 8.22 (6.93-9.52) |
| Delaware | 19.57 (17.93-21.21) | 8.89 (6.55-11.79) |
| District of Columbia | 18.32 (16.24-20.41) | 23.67 (18.06-30.47) |
| Florida | 11.63 (11.38-11.87) | 15.66 (15.01-16.32) |
| Georgia | 13.05 (12.57-13.53) | 11.41 (10.39-12.43) |
| Hawaii | 23.18 (21.79-24.57) | 12.98 (10.61-15.35) |
| Idaho | 14.25 (13.12-15.39) | 13.3 (10.8-15.8) |
| Illinois | 18.09 (17.66-18.52) | 10.87 (10.03-11.7) |
| Indiana | 18.38 (17.77-18.99) | 15.55 (14.15-16.94) |
| Iowa | 17.18 (16.41-17.95) | 15.12 (13.25-16.99) |
| Kansas | 15.76 (14.95-16.57) | 13.32 (11.41-15.23) |
| Kentucky | 26.76 (25.86-27.67) | 29.23 (26.91-31.56) |
| Louisiana | 12.58 (11.96-13.21) | 9.87 (8.53-11.22) |
| Maine | 23.37 (22-24.74) | 11.89 (9.64-14.5) |
| Maryland | 21.18 (20.47-21.89) | 11.38 (10.14-12.62) |
| Massachusetts | 18.31 (17.74-18.87) | 10.81 (9.71-11.9) |
| Michigan | 18.75 (18.27-19.23) | 16.18 (15.06-17.29) |
| Minnesota | 13.99 (13.42-14.57) | 11.9 (10.6-13.19) |
| Mississippi | 18.4 (17.47-19.32) | 19.35 (17-21.7) |
| Missouri | 20.05 (19.42-20.68) | 12.42 (11.17-13.66) |
| Montana | 10.31 (9.21-11.41) | 16.41 (13.14-19.67) |
| Nebraska | 13.77 (12.82-14.71) | 10.94 (8.83-13.06) |
| Nevada | 12.32 (11.45-13.19) | 12.51 (10.64-14.38) |
| New Hampshire | 19.93 (18.56-21.3) | 12.13 (9.76-14.89) |
| New Jersey | 17.6 (17.11-18.09) | 11.33 (10.35-12.32) |
| New Mexico | 14.31 (13.34-15.28) | 13.86 (11.64-16.07) |
| New York | 15.09 (14.79-15.4) | 8.53 (7.96-9.1) |
| North Carolina | 20.79 (20.24-21.34) | 13.99 (12.94-15.04) |
| North Dakota | 23.83 (21.89-25.77) | 20.65 (16.25-25.89) |
| Ohio | 18.45 (18.01-18.88) | 12.62 (11.7-13.53) |
| Oklahoma | 19.44 (18.62-20.26) | 15.33 (13.52-17.13) |
| Oregon | 14.88 (14.2-15.56) | 14.04 (12.44-15.64) |
| Pennsylvania | 17.92 (17.54-18.3) | 12.2 (11.39-13.01) |
| Rhode Island | 30.04 (28.3-31.79) | 16.92 (13.6-20.24) |
| South Carolina | 20.85 (20.07-21.63) | 13.93 (12.48-15.38) |
| South Dakota | 19.26 (17.65-20.86) | 20.53 (16.49-25.26) |
| Tennessee | 25.06 (24.34-25.79) | 20.76 (19.18-22.34) |
| Texas | 20.74 (20.37-21.12) | 19.62 (18.77-20.46) |
| Utah | 11.16 (10.27-12.05) | 9.96 (8.02-11.9) |
| Vermont | 22.8 (20.74-24.87) | 12.22 (8.85-16.46) |
| Virginia | 15.91 (15.37-16.44) | 12.69 (11.58-13.8) |
| Washington | 20.26 (19.61-20.9) | 18.62 (17.16-20.07) |
| West Virginia | 31.65 (30.28-33.02) | 33.34 (29.78-36.9) |
| Wisconsin | 15.68 (15.11-16.26) | 13.77 (12.43-15.12) |
| Wyoming | 15.1 (13.16-17.05) | 18.56 (13.82-24.4) |

Supplemental Table 7: Crude mortality rate per 100,000 associated with AMI and pneumonia stratified by ten-year age group in adults aged ≥65 in the United States, 1999 to 2023

| Crude Rate (95% CI) | | | |
| --- | --- | --- | --- |
| Year | 65-74 years | 75-84 years | 85+ years |
| 1999 | 9.75 (9.29-10.2) | 32.03 (31.03-33.04) | 91.29 (88.38-94.19) |
| 2000 | 8.93 (8.5-9.37) | 31.79 (30.8-32.79) | 97.65 (94.68-100.63) |
| 2001 | 9.01 (8.58-9.45) | 30.24 (29.28-31.2) | 94.52 (91.61-97.42) |
| 2002 | 9.09 (8.66-9.53) | 31.7 (30.73-32.68) | 99.34 (96.39-102.3) |
| 2003 | 8.44 (8.02-8.86) | 28.98 (28.05-29.91) | 96.19 (93.31-99.07) |
| 2004 | 7.58 (7.19-7.97) | 26.3 (25.42-27.18) | 82.05 (79.42-84.69) |
| 2005 | 7.1 (6.72-7.48) | 25.3 (24.44-26.16) | 81.01 (78.43-83.58) |
| 2006 | 6.32 (5.96-6.67) | 22.84 (22.02-23.66) | 73.08 (70.68-75.48) |
| 2007 | 5.94 (5.6-6.29) | 21.06 (20.27-21.84) | 69.23 (66.94-71.53) |
| 2008 | 4.89 (4.59-5.19) | 19.19 (18.44-19.94) | 68.65 (66.4-70.9) |
| 2009 | 4.87 (4.57-5.16) | 17.18 (16.47-17.89) | 58.54 (56.49-60.59) |
| 2010 | 4.79 (4.5-5.09) | 16.99 (16.28-17.7) | 58.38 (56.36-60.4) |
| 2011 | 4.92 (4.63-5.2) | 16.77 (16.07-17.47) | 59.87 (57.87-61.87) |
| 2012 | 4.37 (4.11-4.64) | 15.17 (14.51-15.84) | 53.96 (52.09-55.84) |
| 2013 | 4.39 (4.14-4.65) | 15.42 (14.75-16.08) | 53.2 (51.37-55.04) |
| 2014 | 4.27 (4.02-4.52) | 13.48 (12.86-14.09) | 47 (45.28-48.71) |
| 2015 | 4.24 (4-4.48) | 14.36 (13.73-14.99) | 47.08 (45.38-48.78) |
| 2016 | 4.36 (4.11-4.6) | 12.94 (12.35-13.53) | 44.25 (42.61-45.88) |
| 2017 | 4.21 (3.98-4.44) | 13.07 (12.48-13.65) | 44.35 (42.73-45.98) |
| 2018 | 4.12 (3.89-4.34) | 12.23 (11.68-12.78) | 41.15 (39.59-42.7) |
| 2019 | 3.98 (3.76-4.2) | 11.55 (11.02-12.07) | 36.14 (34.69-37.59) |
| 2020 | 7.14 (6.85-7.43) | 18.28 (17.62-18.93) | 47.29 (45.64-48.95) |
| Total (1999-2020) | 5.77 (5.7-5.83) | 19.94 (19.78-20.1) | 62.54 (62.09-62.99) |
| 2021 | 8.54 (8.22-8.85) | 21.19 (20.48-21.9) | 53.55 (51.69-55.41) |
| 2022 | 6 (5.74-6.26) | 15.36 (14.78-15.95) | 42.2 (40.62-43.78) |
| 2023 | 3.9 (3.69-4.11) | 11.6 (11.11-12.09) | 36.84 (35.32-38.35) |
| Total (2021-2023) | 6.12 (5.97-6.27) | 15.85 (15.51-16.19) | 44.05 (43.1-45.01) |

Supplemental Table 8: Deaths due to AMI and pneumonia, stratified by place of death, in adults of age ≥65 in the United States, 1999 to 2023

| Deaths | | | | | |
| --- | --- | --- | --- | --- | --- |
| Year | Medical Facility | Decedent's Home | Hospice Facility | Nursing Home/Long Term Care | Other |
| 1999 | 8045 | 238 | NA | 1161 | 58 |
| 2000 | 8132 | 269 | NA | 1262 | 48 |
| 2001 | 8092 | 255 | NA | 1123 | 69 |
| 2002 | 8432 | 309 | NA | 1231 | 86 |
| 2003 | 8002 | 286 | NA | 1176 | 95 |
| 2004 | 7041 | 268 | 23 | 1095 | 100 |
| 2005 | 7004 | 281 | 45 | 984 | 102 |
| 2006 | 6435 | 263 | 64 | 901 | 79 |
| 2007 | 6055 | 265 | 99 | 901 | 88 |
| 2008 | 5757 | 258 | 110 | 826 | 73 |
| 2009 | 5202 | 234 | 97 | 694 | 70 |
| 2010 | 5363 | 241 | 112 | 682 | 61 |
| 2011 | 5521 | 253 | 147 | 708 | 104 |
| 2012 | 5022 | 258 | 203 | 650 | 95 |
| 2013 | 5206 | 285 | 203 | 605 | 91 |
| 2014 | 4713 | 280 | 232 | 577 | 56 |
| 2015 | 4970 | 254 | 260 | 572 | 64 |
| 2016 | 4758 | 328 | 274 | 500 | 52 |
| 2017 | 4843 | 296 | 312 | 533 | 52 |
| 2018 | 4655 | 322 | 274 | 504 | 73 |
| 2019 | 4371 | 332 | 289 | 407 | 80 |
| 2020 | 7081 | 451 | 336 | 498 | 106 |
| 2021 | 8178 | 474 | 325 | 425 | 105 |
| 2022 | 6178 | 463 | 315 | 382 | 113 |
| 2023 | 4685 | 374 | 313 | 319 | 74 |
| Total | 153741 | 7537 | 4033 | 18716 | 1994 |

Supplemental Table 9:Sensitive analysis of deaths and age-adjusted mortality rates per 100,000 associated with AMI and pneumonia in adults aged ≥65 in the United States, 1999 to 2023

| Year | Deaths | Population | Age Adjusted Rate | Lower 95% CI | Upper 95% CI |
| --- | --- | --- | --- | --- | --- |
| 1999 | 4997 | 34797841 | 14.58 | 14.17 | 14.98 |
| 2000 | 4938 | 34991753 | 14.21 | 13.81 | 14.6 |
| 2001 | 4722 | 35290291 | 13.41 | 13.03 | 13.79 |
| 2002 | 4926 | 35522207 | 13.8 | 13.41 | 14.18 |
| 2003 | 4638 | 35863529 | 12.8 | 12.43 | 13.17 |
| 2004 | 4088 | 36203319 | 11.14 | 10.8 | 11.48 |
| 2005 | 3918 | 36649798 | 10.49 | 10.17 | 10.82 |
| 2006 | 3498 | 37164107 | 9.2 | 8.89 | 9.5 |
| 2007 | 3305 | 37825711 | 8.48 | 8.19 | 8.77 |
| 2008 | 3231 | 38777621 | 8.09 | 7.81 | 8.37 |
| 2009 | 2926 | 39623175 | 7.23 | 6.97 | 7.49 |
| 2010 | 2849 | 40267984 | 6.87 | 6.62 | 7.12 |
| 2011 | 2945 | 41394141 | 6.89 | 6.64 | 7.14 |
| 2012 | 2759 | 43145356 | 6.3 | 6.07 | 6.54 |
| 2013 | 2747 | 44704074 | 6.11 | 5.88 | 6.34 |
| 2014 | 2523 | 46243211 | 5.48 | 5.26 | 5.69 |
| 2015 | 2563 | 47760852 | 5.45 | 5.24 | 5.66 |
| 2016 | 2460 | 49244195 | 5.08 | 4.88 | 5.29 |
| 2017 | 2488 | 50858679 | 5.05 | 4.85 | 5.25 |
| 2018 | 2353 | 52431193 | 4.65 | 4.46 | 4.84 |
| 2019 | 2193 | 54058263 | 4.23 | 4.06 | 4.41 |
| 2020 | 2310 | 55659365 | 4.36 | 4.19 | 4.54 |
| Total (1999-2020) | 73377 | 928476665 | 7.95 | 7.89 | 8 |
| 2021 | 2313 | 55847953 | 4.54 | 4.35 | 4.72 |
| 2022 | 2246 | 57794852 | 4.15 | 3.98 | 4.33 |
| 2023 | 2044 | 59248361 | 3.79 | 3.63 | 3.96 |
| Total (2021-2023) | 6603 | 172891166 | 4.16 | 4.05 | 4.26 |

Supplemental Figure 1: Overall and sex-stratified AAMRs and APCs associated with AMI and pneumonia in adults aged ≥65 in the United States, 1999 to 2023

* Indicates that the APC significantly differs from zero at α = 0.05.

Supplemental Figure 2: Race-stratified AAMRs and APCs associated with AMI and pneumonia in adults aged ≥65 in the United States, 1999 to 2023

* Indicates that the APC significantly differs from zero at α = 0.05.

Supplemental Figure 3: Urbanization-stratified AAMRs and APCs associated with AMI and pneumonia in adults aged ≥65 in the United States, 1999 to 2020

* Indicates that the APC significantly differs from zero at α = 0.05.

Supplemental Figure 4: Census region-stratified AAMRs and APCs associated with AMI and pneumonia in adults aged ≥65 in the United States, 1999 to 2023

* Indicates that the APC significantly differs from zero at α = 0.05.

Supplemental Figure 5: Age-stratified crude mortality rates and APCs associated with AMI and pneumonia in adults aged ≥65 in the United States, 1999 to 2023

* Indicates that the APC significantly differs from zero at α = 0.05.

Supplemental Figure 6: Sensitive analysis of AAMRs and AAPCs associated with AMI and pneumonia in adults aged ≥65 in the United States, 1999 to 2023

* Indicates that the AAPC is significant.The shaded area represents the 95% CI.

Supplemental Figure 7: Sensitive analysis of AAMRs and APCs associated with AMI and pneumonia in adults aged ≥65 in the United States, 1999 to 2023

* Indicates that the APC significantly differs from zero at α = 0.05.
